# Cold Induces Micro- and Nano-Scale Reorganization of Lipid Raft Markers at Mounds of T-Cell Membrane Fluctuations

**DOI:** 10.1371/journal.pone.0005386

**Published:** 2009-04-30

**Authors:** Yong Chen, Jie Qin, Jiye Cai, Zheng W. Chen

**Affiliations:** 1 Department of Microbiology and Immunology, Center for Primate Biomedical Research, University of Illinois College of Medicine, Chicago, Illinois, United States of America; 2 Institute for Advanced Study, Nanchang University, Nanchang, Jiangxi, China; 3 Department of Chemistry, Jinan University, Guangzhou, Guangdong, China; Université de Toulouse, France

## Abstract

Whether and how cold causes changes in cell-membrane or lipid rafts remain poorly characterized. Using the NSOM/QD and confocal imaging systems, we found that cold caused microscale redistribution of lipid raft markers, GM1 for lipid and CD59 for protein, from the peripheral part of microdomains to the central part on Jurkat T cells, and that cold also induced the nanoscale size-enlargement (1/3- to 2/3-fold) of the nanoclusters of lipid raft markers and even the colocalization of GM1 and CD59 nanoclusters. These findings indicate cold-induced lateral rearrangement/coalescence of raft-related membrane heterogeneity. The cold-induced re-distribution of lipid raft markers under a nearly-natural condition provide clues for their alternations, and help to propose a model in which raft lipids associate themselves or interact with protein components to generate functional membrane heterogeneity in response to stimulus. The data also underscore the possible cold-induced artifacts in early-described cold-related experiments and the detergent-resistance-based analyses of lipid rafts at 4°C, and provide a biophysical explanation for recently-reported cold-induced activation of signaling pathways in T cells. Importantly, our fluorescence-topographic NSOM imaging demonstrated that GM1/CD59 raft markers distributed and re-distributed at mounds but not depressions of T-cell membrane fluctuations. Such mound-top distribution of lipid raft markers or lipid rafts provides spatial advantage for lipid rafts or contact molecules interacting readily with neighboring cells or free molecules.

## Introduction

It has long been recognized that cold/chilling can dramatically induce the morphological change or even activation of human blood platelets[1], [2]. Although not as sensitive to chilling as platelet, other cell types are always questioned as to whether cold casts effects on the ultrastructures in their plasma membranes especially the extensively-studied tiny structure, lipid raft (LR)[3], [4] or membrane raft[5]. It has been reported that GM1/GM3 (two types of lipid raft markers) clusters in plasma membrane of fibroblasts were susceptible to chilling[6]. Cold even induced the activation of signaling pathways by coalescing membrane microdomains on T cells[7]. However, since the cold-induced alternations in plasma membranes are too tiny (at nanoscale) to be detected by conventional fluorescence microscopy, the cold-induced spatial reorganization of lipid rafts or the lateral rearrangement/coalescence of raft-related membrane heterogeneity remains unclear.

Near-field scanning optical microscopy (NSOM) has been used to visualize microdomains or lipid rafts in model membranes[8], [9] or cell membranes[10]–[13]. Recently, we have upgraded the NSOM application in two aspects: i) in combination with fluorescent quantum dot (QD) labeling, the resolution (down to 40 nm) and reproducibility of NSOM imaging has been remarkably improved [14]; ii) nanoscale fluorescence-topographic NSOM imaging has been developed to determine the peak or mound versus depression localization of molecules in cell membrane fluctuations[15].

In this study, we took advantages of our upgraded NSOM imaging and confocal microscopy to precisely visualize and quantify the distribution pattern as well as the cold-induced microscale and nanoscale re-distributions of two types of putative lipid raft markers, GM1 (a lipid marker) and CD59 (a protein maker), to investigate lipid raft-related membrane heterogeneity. In addition, we employed fluorescence-topographic NSOM imaging to determine where lipid raft markers or lipid rafts distribute and redistribute at T-cell membrane fluctuations.

## Results

### Formaldehyde (FA) pre-fixation has distinct effects on the fluorescence staining of GM1 and CD59 in cell plasma membranes

**Figure 1:**
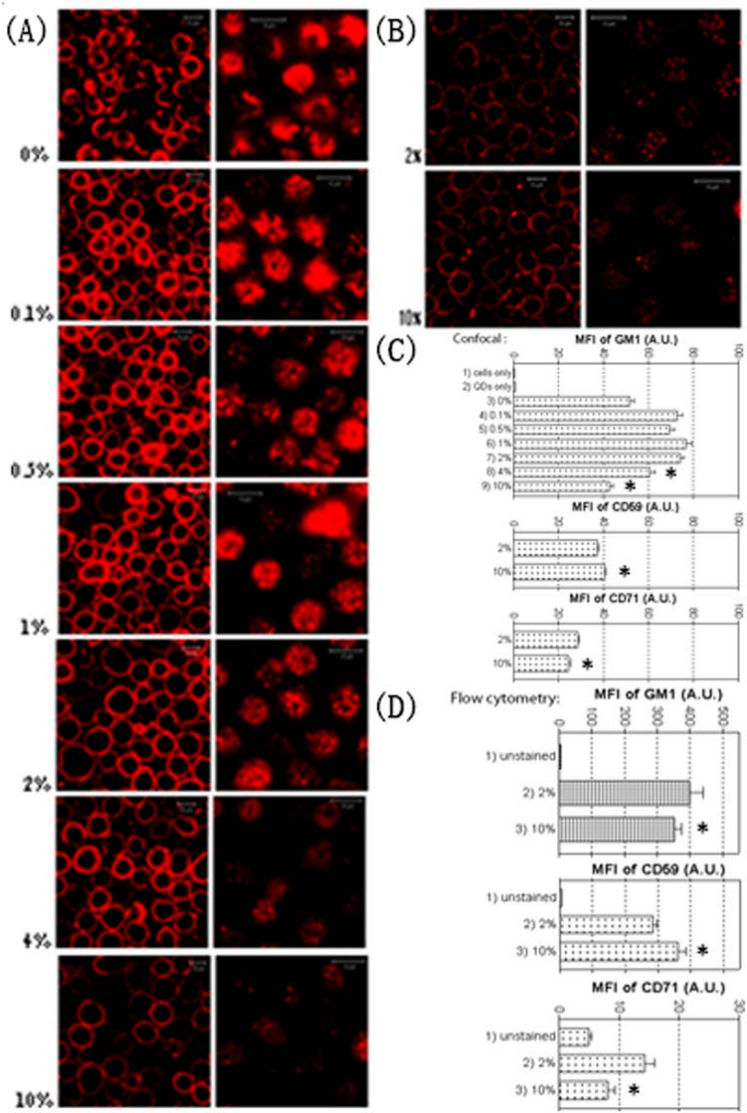
Distinct effects of FA fixation on cell surface fluorescence staining of lipid raft markers on Jurkat T cells. (A) Confocal images of GM1 on Jurkat T cells pre-fixed with or without formaldehyde (FA) of increasing concentrations prior to cell surface staining of biotinylated CTB followed with streptavidin-conjugated QD655. Left/right panel: focus is on the central/surface stack of the same cells. It is evident that the fluorescence staining of GM1 on 10% FA-fixed cells is much worse than other groups. (B) Confocal images of CD59 on Jurkat T cells pre-fixed by 2% or 10% FA, showing a better fluorescence staining of CD59 on 10% FA-fixed cells. (C) Mean fluorescence intensity (MFI) of the central stacks of individual cells (∼200 cells/group in the first graph; ∼350–450 cells/group in graphs 2 and 3) imaged by confocal microscopy under various fixation conditions. (D) MFI of whole cells detected by flow cytometry (Mean±SEM). Here, FITC-conjugated CTB, anti-CD59, and anti-CD71 were used to exclude the possibility that the distinct effects of fixation were caused by its effects on the labeling or fluorescent properties of QD dyes. (All procedures done at 4°C).

Since imaging studies of cold-induced effects on lipid rafts require formaldehyde (FA) pre-fixation for immune staining of lipid raft-enriched Jurkat T cells, we first examined effects of FA on the fluorescence staining of various types of molecules in plasma membranes. Surprisingly, we found that FA pre-fixation posed significant effects on GM1 (a lipid marker of LR), CD59 (a protein marker of LR), and CD71 (transferrin receptor, a non-raft protein). The confocal images of GM1 on Jurkat T cells pre-fixed with different concentrations of FA showed that the fluorescence staining of GM1 on cell surface was evidently impaired when FA concentrations increased to 4–10% (Fig. 1A). The results were confirmed by mean fluorescence intensity (MFI) analyses of the cells (first panel of Fig. 1C), and consistent with the flow cytometric data (first panel of Fig. 1D). In contrast, higher concentration (e.g. 10%) of FA enhanced the fluorescence staining of CD59 on cell surfaces compared to 2% FA (Fig. 1B, second panels of Figs. 1C and 1D). Interestingly, however, the effect of FA fixation on the fluorescence staining of CD71, a non-raft protein, was similar to GM1 as indicated by MFI analyses (third panel of Fig. 1C), and flow cytometric data (third panel of Fig. 1D). Thus, FA pre-fixation has distinct effects on the fluorescence staining of GM1, CD71, and CD59 in cell plasma membranes.

### A MFI ratio-based method for evaluating the effects of fixation or cold on the re-distribution of molecules within individual microdomains under confocal microscopy

**Figure 2:**
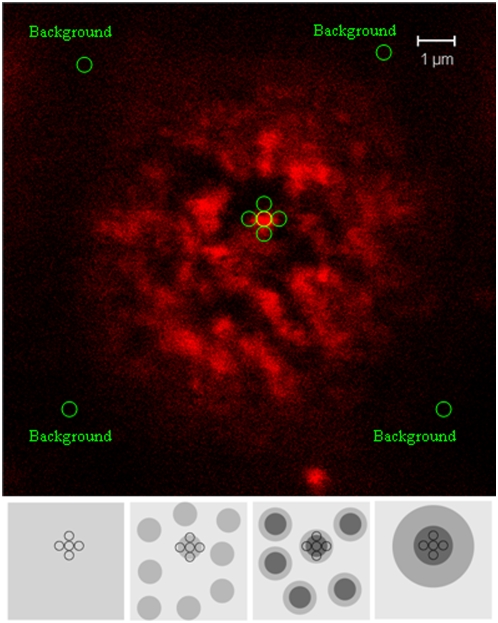
The MFI ratio-based method for evaluating the effects of fixation or cold on the redistribution of molecules or nanoclusters of lipid raft markers within individual microdomains by confocal. Upper panel: the upper surface/stack of a Jurkat T cell is shown here, on which many microdomains are evident. In the MFI ratio-based method, each microdomain was divided into central and peripheral parts, and the measurement and calculation of MFI ratio of microdomains are as follows: A: MFI of the central circle (∼0.29 um^2^; radius = ∼300 nm) on the center of each microdomain; B: Mean of the MFIs of the peripheral four circles on the peripheral areas of each microdomain; C: Mean of the MFIs of the background (generally 7 circles). MFI of the central part of each microdomain = A–C; MFI Ratio of each microdomain = (A–C)/(B–C). Bottom panel: the schematic diagram shows the distribution alternations of microdomains. The left and most-right graphs show the two potential scenarios for the MFI ratio to be very close to 1.0: (left graph) the molecules or nanoclusters distribute uniformly on the whole cells, therefore actually there are no confocal-detectable microdomains on cell surfaces or the whole cell membrane can be regarded as a single microdomain; (most-right graph) the microdomains have a very big size (>> 2 μm), in the central part of which the molecules or nanoclusters distribute uniformly. The two middle graphs show that the increase of MFI ratio indicates the increase of the molecule amounts in the central part of individual microdomains compared with the molecule amount in the peripheral part of the same microdomains, therefore implying the redistribution of molecules or clusters from peripheral part to central part within individual microdomains.

To quantitatively analyze and compare the fixation-related or cold-induced distribution changes of GM1 or CD59 within individual microdomains, we developed a MFI ratio-based method using confocal imaging since a size/intensity analysis of individual microdomains can not evaluate the difference between different parts within individual microdomains. For this method, individual GM1 or CD59 microdomains on cell surface were artificially divided into two parts: central and peripheral parts (Fig. 2). Here, MFI ratio is defined by the formulation that the MFI of central part (an area of ∼0.29 um^2^; radius = ∼300 nm) minus MFI of background divides the mean MFI of peripheral part (i.e., the mean value of the MFIs of four areas surrounding central part) minus MFI of background. An increase in MFI ratio indicates an increased molecule amount in the central part of individual microdomains compared to the molecule amount in the peripheral part of the same microdomains. Therefore the change of MFI ratio also implies the re-distribution of molecules or nanoclusters from peripheral part to central part within individual microdomains. When the MFI ratio is getting very close to 1.0, there are two scenarios: i) the molecules or nanoclusters distribute uniformly on the whole cells, therefore actually there are no confocal-microscopy-detectable microdomains on cell surfaces or the whole cell plasma membrane can be regarded as a single microdomain; ii) the microdomains are very big (>> 2 μm) in which molecules or nanoclusters uniformly distribute.

### 2% FA pre-fixation prevents the re-distribution of GM1 from the peripheral part to the central part of microdomains but allows them to move from outside to the peripheral part

**Figure 3:**
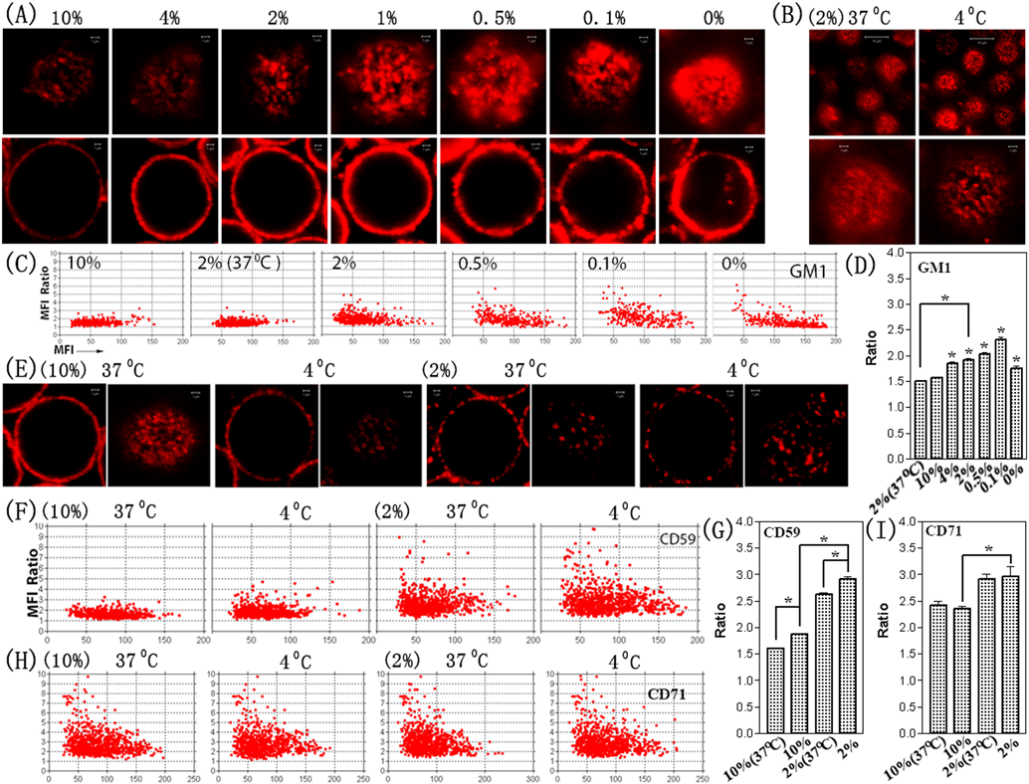
Confocal microscopy coarsely visualizes and quantifies the distinct effects of fixative on lipid raft markers on Jurkat T cells. (A) shows confocal images of GM1 on individual Jurkat T cells pre-fixed without or with increasing concentrations of FA as shown at Fig. 1A. Cells in left panel were the same cells in right panel, focusing on the central parts (left panel) or upper surfaces (right panel) of the cells. (B) shows confocal images of GM1 on cells pre-fixed with 2% FA at 4°C or 37°C. (C, F, H) The Ratio-versus-MFI dot graphs quantify the crosslinking- and cold-induced redistributions of GM1 (C; 20 cells/group), CD59 (F; 25 cells/group), and CD71 (H; 15 cells/group) microdomains on cell surface (the data of all measured microdomains on every cell were displayed in the graphs). (E) shows confocal images of CD59 on individual cells pre-fixed with 2% or 10% FA at 4°C or 37°C. (D, G, I) The corresponding histograms (Mean±SEM) quantifies the cold-induced redistributions of them at cell surfaces, respectively. Mark “*” shows the significant difference (P<0.01).

Fig. 3A shows the confocal images of GM1 microdomains on Jurkat T cells pre-fixed by10%, 4%, 2%, 1%, 0.5%, 0.1%, or 0% FA at 4°C for 30 min, and subsequently stained by biotinylated Cholera Toxin subunit B (CTB) and QD655-conjugated streptavidin. Evidently, the microdomains became larger and brighter due to the decrease in fixation strength and the cross-linking effects of the staining reagents, implying a significant enrichment of GM1 molecules or nanoclusters into microdomains or a fusion among microdomains.

Fig. 3C shows the alternations of the MFI ratio (Y axes) and the MFI of the central part (X axes) of all individual GM1 microdomains (each dot represents a GM1 microdomain in the dot graphs) on upper surface of 20 cells in each group under various fixation conditions. An increase in the MFI ratio of GM1 microdomains was evident when the fixation strength decreased: 1.56±0.29 (Mean±SD; 10% FA), 1.85±0.59 (4%), 1.92±0.52 (2%), 2.03±0.65 (0.5%), and 2.31±0.86 (0.1%), respectively (P<0.0001 for each pairs; Fig. 3D). The drop of MFI ratio (1.75±0.75; Mean±SD) in the no-fixation (0% FA) group was due to the formation of many large-size GM1 microdomains as reasoned above.

Interestingly, there were no significant changes (P = 0.157) in the mean MFI of the central part of GM1 micoromains between the 2% (63.9±27.8) and 10% (61.3±25.4) groups, implying that less GM1 molecules or nanoclusters entered into the central part of the domains from the peripheral part. Based on the data from the MFI ratio and the MFI of the central part of microdomains, we concluded that the 2%-FA fixation stopped the redistribution of GM1 from the peripheral part of microdomains to the central part but did not completely stop the redistribution from outsides into the peripheral part of microdomains.

### 2% FA pre-fixation could not completely stop the redistribution of CD59 and CD71 from the peripheral part to the central part of microdomains

Similarly, a dramatic decrease (P<0.0001) in MFI ratio of CD59 microdomains from 2.92±1.24 (Mean±SD) down to 1.87±0.50 was detected when the fixation strength was enhanced from 2% to 10% FA. The mean MFI of the central part of CD59 microdomains slightly but significantly (P<0.0001) decreased from 82.2±33.5 (Mean±SD) to 66.0±24.1. The data implies that 2% FA fixation could not stop a slight redistribution of CD59 from the peripheral part to the central part of CD59 microdomains or a dramatic redistribution from the outside to the peripheral part. In another word, 10% FA had much better efficiency for fixing CD59 than 2% FA. Since 10% FA fixation also enhanced the fluorescence staining of CD59 (Fig. 1B–1D), the 10%-FA fixation condition was used for subsequent NSOM studies of cold-induced changes in CD59.

A weaker but still significant change (P<0.01) in MFI ratio of CD71 microdomains also occurred between 2%- and 10%-FA fixation groups. However, the 4°C/37°C temperature change did not significantly alter the MFI ratio (P = 0.774 and P = 0.497 for 2%- and 10%-FA fixation group, respectively) of CD71 microdomains (Figs. 3H, 3I). The mean MFI of the central part of CD71 microdomains also significantly (P<0.0001) decreased from 82.3±38.1 (Mean±SD; 2% FA) to 64.1±25.5 (10% FA) to the similar extent as CD59.

### The 2% FA fixation strength is enough for preventing the clustering of GM1 molecules or nanoclusters

**Figure 4:**
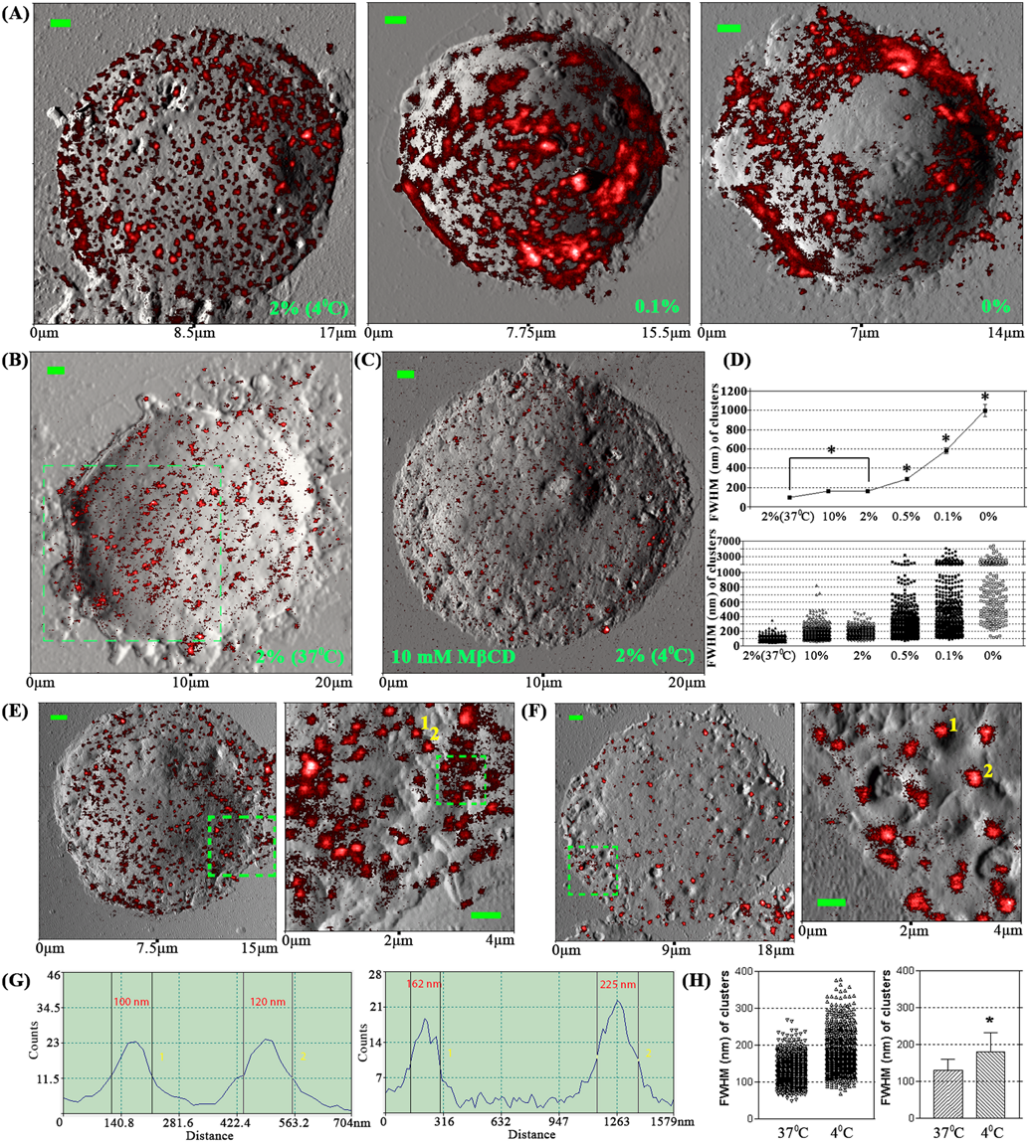
Accurate NSOM imaging and quantification of GM1/CD59 on Jurkat T cells pre-fixed by FA of various concentrations and at various temperatures. (A) NSOM images (merged from the NSOM fluorescence images in red and the corresponding topographic images in gray) show the size enlargement of GM1 clusters from nanoclusters to microclusters with the decrease in fixation strength from 2% FA to 0% no fixation. (B, C) The NSOM images of GM1 from representatives of Jurkat cells pre-fixed by 2% FA at 37°C (B) or treated with 10 mM MβCD for 30 min followed by pre-fixation with 2% FA at 4°C and fluorescence staining (C). (D) Dot graph (bottom) and X-Y plot (top; SEM±SD) of the diameters (FWHM) of the GM1 micro/nanoclusters on cells (5 cells/group) pre-fixed with 0%, 0.1%, 0.5%, 2%, 10% FA at 4°C, and 2% FA at 37°C, respectively. (E, F) NSOM images of CD59 on a representative cell (Left) pre-fixed by 10% FA at 4°C (E) or at 37°C (F) and the boxed part (right) of it; (G) The fluorescence profiles of the cross sections across the centers of the two CD59 nanoclusters indicated by numbers 1 and 2 in Fig. E (left of Fig. G) or F (right). (H) Dot (left) and bar (right; Mean±SD) graphs of the diameters (FWHM) of the CD59 nanoclusters on cells (5 cells/group) pre-fixed with 10% FA at 4°C and at 37°C, respectively). Scan size: (A: left to right) 14×14 μm^2^; 15.5×15.5 μm^2^; 17×17 μm^2^. (B, C) 20×20 μm^2^; (E: left to right) 18×18 μm^2^; ∼4×4 μm^2^; (F: left to right) 15×15 μm^2^; ∼4×4 μm^2^. Scale bar/resolution: (A–C, and left panels of E and F) 1 μm/500×500 pixel^2^; (right panels of E and F) 500 nm/300×300 pixel^2^. Integration time: (all) 30 ms.

High resolution NSOM was then used to visualize and quantify the increase in size (from nanoclusters to microclusters) in the presence of enhanced cross-linking (or impaired fixation): 165.0±49.0 nm (Mean±SD; 2% FA), 287.8±241.7 nm (0.5%), 580.3±644.8 nm (0.1%), and 999.1±925.1 nm (0%), respectively (P<0.0001 for each pairs; Fig. 4A, 4D). Interestingly, we found that the size (163.8±73.3 nm; Mean±SD) of GM1 nanoclusters on cells pre-fixed by 10% FA was similar (P = 0.6153) to that (165.0±49.0 nm) on cells pre-fixed by 2% FA (Fig. 4D). It implied that 2%-FA fixation was sufficient for preventing the clustering of GM1 molecules or nanoclusters although there were many GM1 clusters that tended to approach the confocal-microscopy microdomains containing nanoclusters of various sizes. Since 10%-FA fixation dramatically impaired the fluorescence staining of GM1 (Fig. 1A, 1C, 1D), the 2%-FA fixation condition was used for the subsequent NSOM studies of cold-induced changes in GM1.

### Cold induces the microscale centralization of GM1 or CD59 within individual microdomains, and the nanoscale coalescence of GM1/CD59 nanoclusters

The confocal data showed that the MFI ratio of GM1 (Figs. 3C, 3D) or CD59 (Figs. 3F, 3G) not CD71 (Figs. 3H, 3I) increased at a cold temperature (4°C) compared to the physiological temperature (37°C). The MFI ratio of GM1 microdomains on cells pre-fixed by 2% FA increased from 1.51±0.29 (37°C) to 1.92±0.52 (4°C) due to chilling (Fig. 3D); the MFI ratio of CD59 microdomains (Fig. 3G) on cells fixed by 2% FA increased from 2.63±0.94 (37°C) to 2.92±1.24 (4°C); the MFI ratio of CD59 microdomains on cells fixed by 10% FA increased from 1.61±0.31 (37°C) to 1.87±0.50 (4°C). All P values of them are less than 0.0001. The data implies that cold induced a microscale redistribution or centralization of lipid rafts within individual microdomains.

NSOM images showed that the nanoclusters of GM1 or CD59 at 4°C (left panel of Fig. 4A for GM1; Fig. 4F for CD59) were larger than those at 37°C (Fig. 4B for GM1; Fig. 4E for CD59). Quantitative analyses indicate that chilling induced a nanoscale enlargement (1/3–2/3 time; P<0.0001) of the mean size of the nanoclusters from 97.8±30.0 nm (37°C) to 165.0±49.0 nm (4°C) for GM1 (Fig. 4D), or from 130.1±29.4 nm (37°C) to 179.7±52.1 nm (4°C) for CD59 (Figs. 4G, 4H). The method of size analysis has been described previously[15], and all NSOM-detectable features were counted. The very irregular shapes shown in the NSOM images were the minority features (<5% of total features), and therefore, their sizes did not affect the analytical result although we treated the irregular shapes as a round shape for analytical convenience. The dramatic size decrease of GM1 nanoclusters induced by the treatment of 10 mM MβCD (a cholesterol-depletion reagent) for 30 min prior to fixation implies the cholesterol-sensitivity/association of lipid rafts (see Fig. S1 in Supplementary materials for related confocal images).

### NSOM visualizes the distribution of GM1/CD59 on fixed cells in a nearly-natural state of live cells

2% FA at 37°C (physiological temperature) for GM1 and 10% FA at 37°C for CD59 appeared to be the best conditions for fixing the two types of lipid raft markers, which minimized their moving from the peripheral parts to the central parts of GM1/CD59 micordomains and the clustering or enlarging of the molecules or nanoclusters, although it is impossible to completely avoid the effects of FA fixation on them. Thus, under these best conditions, GM1/CD59 molecules or nanoclusters on the fixed cells are very close but not identical to their natural or original status on live cells. We then sought to image the organization of GM1/CD59 in this nearly-natural state by high-resolution NSOM.

**Figure 5:**
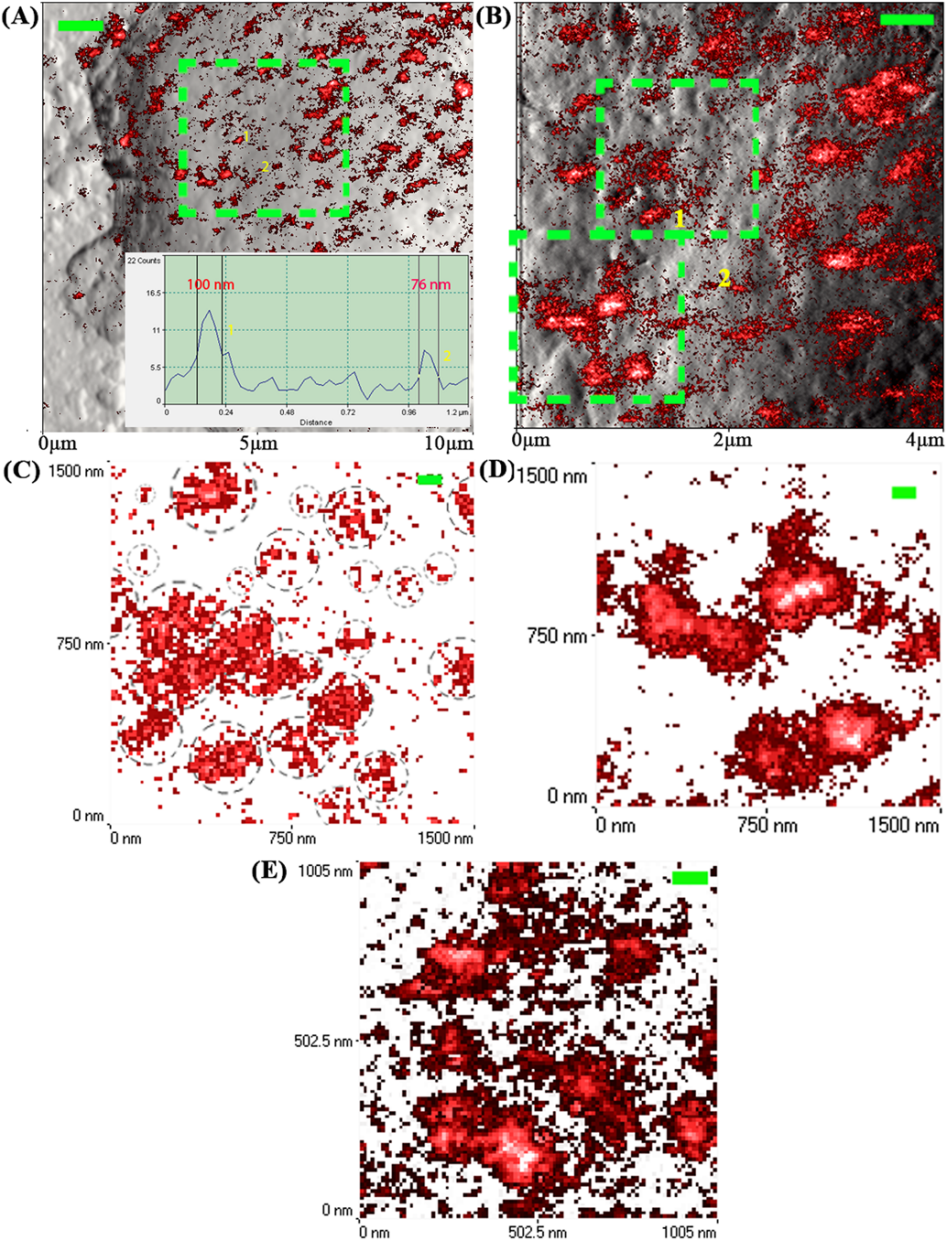
Direct NSOM visualization of the distribution of lipid raft markers at nearly-natural state under best fixation conditions. (A) The NSOM image of GM1 (merged from the NSOM fluorescence images in red and the corresponding topographic images in gray) was magnified from the boxed area on the cell in Fig. 4B (Inset: the fluorescence profile of the cross section across the center of the two nanoclusters indicated by numbers 1 and 2). (B) The NSOM image was magnified from the boxed area in Fig. A. (C, D) The NSOM images, corresponding to the upper (C) and lower (D) boxed areas in Fig. B, were merged from three (C) and four (D) repeatedly-scanned fluorescence images of the same areas to balance the blinking effect of fluorescent QD. (E) The NSOM image of CD59 (merged from three repeatedly-scanned fluorescence images of the same area) was magnified from the boxed area on the cell in the right image of Fig. 4E. Scan size: (A–E) 10×10 μm^2^; 4×4 μm^2^; 1.5×1.5 μm^2^; 1.5×1.5 μm^2^; ∼1.0×1.0 μm^2^. Scale bar/resolution: (A) 1 μm/500×500 pixel^2^; (B) 500 nm/400×400 pixel^2^; (C–E) 100 nm/100×100 pixel^2^; Integration time: (all) 30 ms.

In order to balance the blinking effects of fluorescent QD (on/off switching of QD fluorescence)[14] and weaken the interference of randomly distribution background, a series of 3 or 4 NSOM fluorescence images were taken by repeatedly scanning a same area (1.5 μm×1.5 or ∼1 μm×1 μm) on cell surfaces and were merged into one image. Quantitative analysis (Fig. 5) showed a mean GM1 nanocluster size of 97.8±30.0 nm (Mean±SD) ranging from 44 nm to 164 nm (Fig. 4D) and a mean CD59 nanocluster size of 130.1±29.4 nm ranging from 48 nm to 269 nm (Fig. 4H). Clearly, the distribution of GM1/CD59 was not uniform (Fig. 5). Many GM1/CD59 nanoclusters of various sizes (<100 nm) were loosely confined within individual nanoscale regions (<200 nm; the boundaries of the regions are indicated by the dashed circles in Fig. 5C), which were surrounded by randomly-distributed smallest nanoclusters or maybe single-QD-bound molecules. Individual nanoscale regions have the trend to form single denser nanoclusters by concentrating the small nanoclusters within or surrounding the nanoscale regions, or to form large nanoclusters by aggregating adjacent nanoscale regions (Figs. 5D, 5E).

### Two-color NSOM visualizes the cold-induced colocalization of GM1 and CD59

By evaluating the colocalization status of GM1 with CD59 or CD71 on the same cells with or without treatments of GM1-crosslinking, CD59-crosslinking, and PHA-stimulating, we found that, under relatively-low-resolution confocal microscopy, GM1 microdomains always colocalized with CD59 not CD71 microdomains except that both CD59 and CD71 microdomains stayed together with GM1 microdomains upon PHA stimulation due to the cross-linking effect of PHA (Fig. S2 in Supplementary materials). Here, the colocalization statue of GM1 and CD59 was at the level of microdomain but not nanocluster under the confocal. Since confocal was unable to detect the colocalization of individual nanoclusters or lipid rafts, we used higher-resolution, two-color NSOM to investigate the colocalization of GM1 and CD59 and to see if chilling had impact on the co-localization of them.

**Figure 6:**
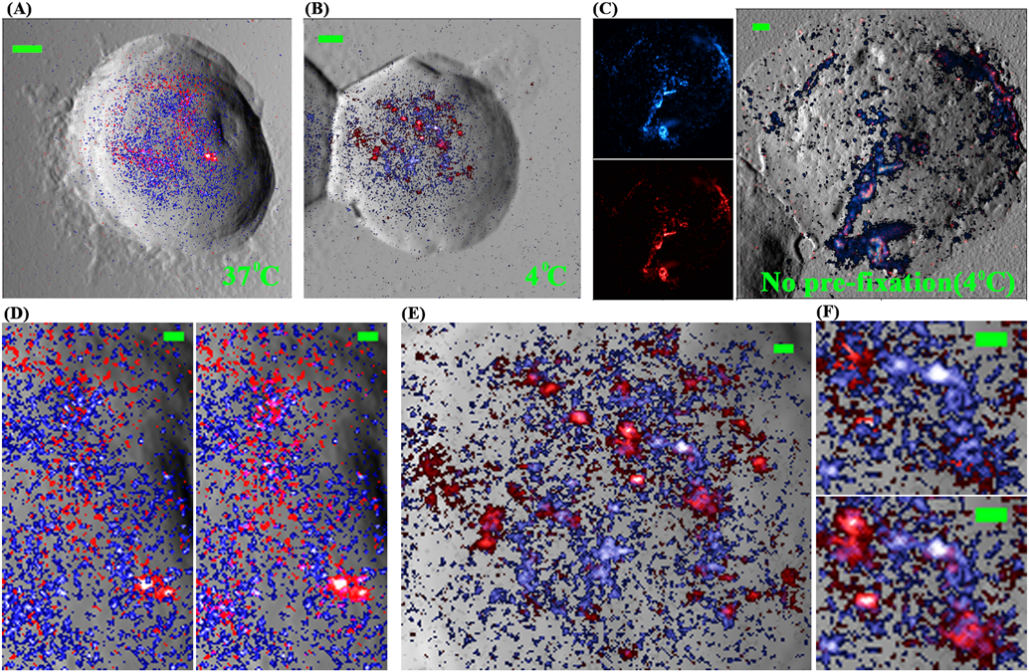
Two-color NSOM directly visualizes the cold-induced colocalization of GM1 and CD59 nanoclusters. (A–C) NSOM-imaged distributions of GM1 (psudocolor: blue; streptavidin-conjugated QD605) and CD59 (red; goat anti-mouse IgG-conjugated QD655) on Jurkat T cells pre-fixed without (C) or with 2% FA at 4°C (B) or at 37°C (A) prior to cell surface staining. (D), (E) and (F) were enlarged from (A), (B), and (E), respectively, showing the details of the distribution/redistribution. In the left panels of (D, E) and the upper panel of (F), the blue color is above the red color; in the right panels of (D, E) and the lower panel of (F), the two colors were merged to show the nanoclusters in which the two raft marker types co-localize (pink). Scan size: (A) 10×10 μm^2^; (B) 11×11 μm^2^; (C) 18×18 μm^2^. Scale bar: (A–C) 1 μm; (D–F) 200 nm. Resolution: (A–C) 500×500 pixel^2^. Integration time: (all) 30 ms.

The cells were pre-fixed with or without 2% FA at 4°C or 37°C, stained by GM1 reagents, fixed second-time, stained by anti-CD59, fixed last time, and then imaged by two-color NSOM. Here, multiple fixation steps were utilized to reduce the disassociation of reacted reagents/antibodies/QDs from cell surfaces during multiple washes of the samples. No significant effects of post-fixation treatments on cell surface staining and membrane structures (or GM1/CD59 distribution) were detected according to our study (data not shown). Consistent with the confocal data (Fig. S2 in Supplementary materials), the microclusters (hundreds of nanometer or even >1 μm) of GM1 and CD59 almost overlaid with each other in the membranes of non-prefixed cells (Fig. 6C).

Interestingly, we found that at physiological temperature the majority of the tiny GM1 and CD59 nanoclusters (∼100 nm or smaller) on cells pre-fixed by 2%-FA did not overlay/overlap with each other although most of them were neighboring/adjacent (Figs. 6A, 6D). However, after chilling from 37°C to 4°C, most GM1 and CD59 nanoclusters colocalized (Figs. 6B, 6E, 6F) with each other. The colocalization manner was that several smaller nanoclusters in one of the two types of lipid raft markers distributed in or around a relatively-larger nanocluster of the other type of raft marker (Fig. 6F). The data implies that cold induced or enhanced the redistribution and colocalization of GM1 and CD59.

Our experiments have to be performed using fixed cells due to the limitations of the reagents and the equipment. Labeling raft antigens, either by antibodies or toxins, always results in some degree of clustering[16]. Due to the experimental limitations, the plasma membrane of fixed cells differs more or less from that of live cells. Under the best fixation conditions, the membrane features of fixed cells were kept closest but not identical to their natural state of live cells. Actually, just like snapshots, the fixation treatments capture the various moments of lateral rearrangement of membrane heterogeneity and other dynamic processes. To some extent, the co-existence of the GM1/CD59 nanoclusters of various sizes and the nanoscale regions confining small GM1/CD59 nanoclusters on the same plasma membrane planes on the fixed cells under the best fixation conditions reflects some degree of the live cell phenomenon.

### Fluorescence-topographic NSOM visualizes the localization and redistribution of GM1/CD59 mainly at the mounds of T-cell membrane fluctuations

**Figure 7:**
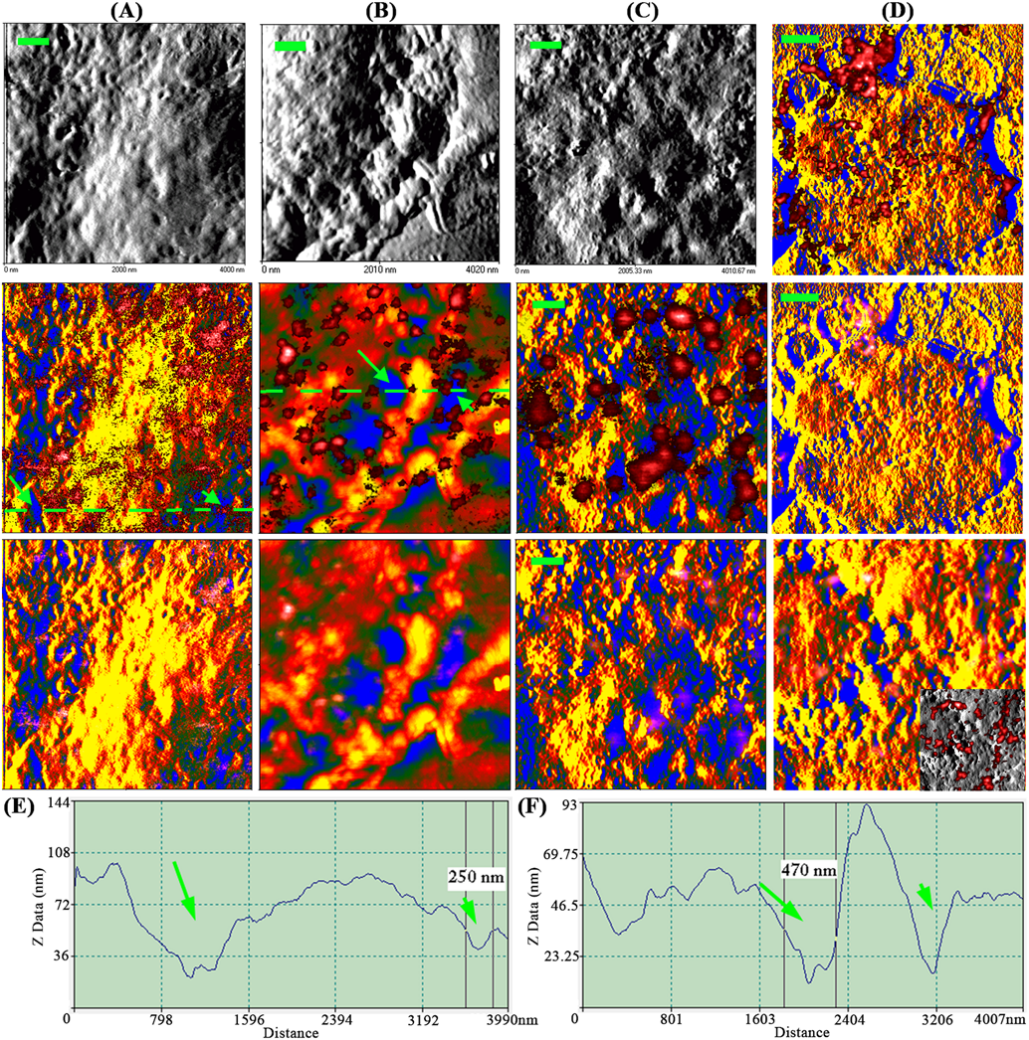
Fluorescence-topographic NSOM directly visualizes the location/distribution and cold- or crosslinking-induced re-distribution of GM1/CD59 nano/microclusters at the mounds not depressions of plasma membrane fluctuations of Jurkat T cells. (A, C, D) NSOM images of GM1 on membrane surface of Jurkat cells pre-fixed without (D) and with 2% FA at 4°C (C) or 37°C (A). (B) NSOM images of CD59 on membrane surface of Jurkat cells pre-fixed with 10% FA at 37°C. (A–C) The upper panels show the NSOM topographic images in gray; In the middle and bottom panels, the topographic information was pseudo-colored for the mound of membrane protrusions in yellow and for the planar membrane or the depression between membrane protrusions in blue; In the middle fluorescence-topographic images, the fluorescence information (in red) was above but not merged with the topographic information (in yellow/blue), highlighting all nanoclusters in red; In the bottom fluorescence-topographic images, the topographic and fluorescence information was merged, highlighting the depression-localizing nanoclusters in pink. Scale bar: 500 nm. (D) Upper: fluorescence information is above but not merged with the pseudo-colored topographic information; middle: fluorescence information is merged with the pseudo-colored topographic information; lower: color-merged image enlarged from the area indicated by the dashed box (Inset: the corresponding fluorescence-topographic image in which the topographic information in gray is not color-merged with the fluorescence information in red). Scale bar: 2 μm. (E, F) Topographic profiles of the cross sections along the dashed lines in the middle images of panels A (E) and B (F). Long arrows show the large depressions (≥500 nm), in which there are no GM1/CD59 nanoclusters. A few GM1/CD59 nanoclusters also distribute, at least partially, in smaller depressions (≤300 nm) shown by short arrows.

Recently, we have developed the nanoscale fluorescence-topographic NSOM imaging method to detect the localization of membrane molecules on cell membrane fluctuations through combining fluorescence molecule information and topographic information in local membrane fluctuations[15]. Here, we used this method to elucidate the localization of GM1 and CD59 at plasma membrane fluctuations of Jurkat T cells. Interestingly, in a nearly-natural state, both GM1 and CD59 nanoclusters mainly localized at the mound not depressions of the plasma membrane fluctuations (Figs. 7A and 7B). After chilling (Fig. 7C) or reagent/antibody-induced crosslinking of GM1 (Fig. 7D), the nanoclusters or microclusters still stayed mainly at the mound sites of membrane fluctuations.

Generally, the outer diameter of a NSOM probe tip is around 250 nm. Therefore, a NSOM probe tip can not enter a small depression with an entry of less than 250 nm in diameter. However, the results show that there are no GM1/CD59 nanoclusters in most large depressions (diameter >300 nm) as shown by the long arrows (Figs. 7E, 7F) and the large blue areas (Figs. 7A–7D). On the other hand, the optical information from a location < 100 nm away from the NSOM probe tip can also be detected, although the information gradually weakens with the increase of distance. This suggests that molecules/nanoclusters in a small depression whose depth is less than 100 nm are likely detectable, even though NSOM probe tips do not enter the depressions. The GM1/CD59 nanoclusters in a few small depressions whose diameter is less than 300 nm (indicated by the short arrows in Figs. 7E, 7F) were still detected by NSOM probe tips. It is possible that the sensitivity to detecting GM1/CD59 in the lower membrane regions, whose diameter is less than 250 nm and depth is more than 100 nm, may be little lower. However, smaller depressions are shallower. The topographic NSOM images and cross sections (Figs. 7E, 7F and data not shown) show that almost all depressions have a depth of less than 100 nm and that most depressions with a diameter of less than 300 nm have a depth of less than 50 nm. Therefore, our study does not significantly underestimate GM1/CD59 in the lower membrane regions.

## Discussion

It is well known that reagent/antibody-induced cross-linking of a lipid raft component causes dramatic clustering of lipid rafts on living cells[17], [18] or phase separation in model membranes[19]. To avoid the reagent/antibody-induced cross-linking, cells were fixed by formaldehyde (FA) prior to fluorescence staining. Therefore, we first performed experiments to see the effects of FA on the fluorescence staining and the organization of two types of putative lipid raft markers (GM1, a lipid marker, and CD59, a protein marker).

The data indicated that 10%-FA pre-fixation dramatically impaired the fluorescence staining of GM1, and that 2%-FA pre-fixation was strong enough for preventing the microscale redistribution of GM1 from the peripheral part of GM1 microdomains to the central part and the nanoscale clustering of GM1. Therefore, 2%-FA pre-fixation was utilized for the studies of cold-driven changes in GM1. In contrast, 2%-FA pre-fixation could not completely prevent the redistribution of CD59 from the peripheral part of CD59 microdomains to the central part, and the fixation of higher-concentration (10%) FA significantly enhanced the fluorescence staining of CD59. Based on this, 10%-FA pre-fixation was used for the studies of cold-induced changes in CD59 nanoclusters.

We found that GM1/CD59 nanoclusters of various sizes (the smallest nanoclusters may be single molecules since it is hard for NSOM to distinguish single molecules from single nanoclusters of tens nanometer) co-exist in the same membrane planes at nearly-natural state (under the best fixation conditions). And piles of nanoclusters of various sizes are loosely confined within individual nanoscale regions (<200 nm) surrounded by randomly-distributed smallest nanoclusters. This distribution pattern of lipid raft markers at nearly-natural state is likely the early intermediate stage between the even distribution at natural state and the cold-/crosslinking-induced uneven distribution.

Interestingly, after chilling the redistribution of the raft lipid/protein markers GM1/CD59 occurred at two levels: their microscale centralization within individual microdomains, and the nanoscale coalescence of nanoclusters with a 1/3–2/3 size increase (from 97.8±30.0 nm to 165.0±49.0 nm for GM1, or from 130.1±29.4 nm to 179.7±52.1 nm for CD59). In contrast, cold did not induce a significant redistribution of the control non-raft marker CD71. Our results imply that membrane chilling reveals the self-associative properties of raft lipids and their interactions with raft proteins, as proposed in model membranes[20]. Surprisingly, two-color NSOM indicated that cold even induces the colocalization of GM1 and CD59. These findings help to propose a model in which the self-associative raft lipids associate themselves and interact with protein interactions to generate functional membrane heterogeneity. On the other hand, the cold-induced coalescence and colocalization of lipid raft markers provide implication that strict liquid-ordered (Lo)/liquid-disordered (Ld) phase transitions do not holistically account for raft-related membrane heterogeneity on live cells[21]. Chilling also increases the propensity conferring membrane condensation and lateral separation of more ordered from less ordered membrane heterogeneity.

In most reported studies on lipid rafts, a detergent resistance-based biochemical technique is used to extract plasma membrane in the nonionic detergent Triton X-100 at 4°C, and thus to sort soluble and insoluble components via sucrose density gradient centrifugation. This most widely-used method makes it possible to demonstrate the existence, residence, and co-localization of lipid raft markers. However, this method has long been argued and controversial [22]–[25]. The domain-inducing effects of detergents on lipid rafts have extensively been studied [26]–[28]. The reduction in temperature from 37°C to 4°C is also suspected to potentially induce alternations in the organization of lipid rafts[6], [22]. To date, the cold-induced nanoscale morphology of lipid rafts that leads to potential changes in cellular function remains unknown. Our data provides direct evidence for the potential temperature-related artifact in the widely-used, detergent resistance-based biochemical assay that operates at 4°C. Generally, the redistribution or rearrangement of lipid raft-resident components may trigger the occurring of some related or downstream events[19], [29], [30]. Therefore, it is not surprising that the cold-induced microscale and nanoscale redistribution or colocalization of lipid raft markers causes the activation of raft-related[31] signaling pathways in T cells[7].

Where lipid rafts localize and where lipid raft-related events occur at membrane fluctuations are important questions. However, due to a lack of useful techniques, these questions are poorly addressed. Here, using fluorescence-topographic NSOM imaging, we found that GM1/CD59 localized at the mounds of membrane fluctuations of Jurkat T cells. Interestingly, our recent study showed that GM1 localizes at the depressions of membrane fluctuations on polarized MDCK cells[15]. However, the fact that GM1 generally are not expressed on MDCK cells implies that GM1 has no function on MDCK cells (or GM1 is a resting molecule). But GM1 is an active molecule on T cells. The data provides an implication that resting molecules or rafts are hidden at depressions of membrane fluctuations and active molecules or rafts are exposed at mounds/peaks of membrane fluctuations for easier reaching the molecules or rafts on neighboring cells for cell-cell interaction. However, further studies are needed to support this hypothesis.

## Materials and Methods

### Cell Culture and Reagents

Jurkat (E6-1) T cell line was purchased from ATCC. Cells were cultured in RPMI-1640 medium supplemented with 10% heat-inactivated fetal bovine serum (FBS), 2 mM L-glutamine, 1% sodium pyruvate, and basal medium eagle (BME). Cell cultures were maintained at a cell concentration between ∼5×10^5^ viable cells/ml and refreshed every 2–3 days depending on cell density.

The reagents/antibodies used are as follows: biotinylated CTB, FITC-conjuaged CTB, PHA, and MβCD were purchased from Sigma-Aldrich (St Louis, MO, USA); fluorescent QD655-conjugated streptavidin, QD605-conjugated strepavidin, and QD655-conjugated goat anti-mouse IgG antibody (H+L) were from Invitrogen (Carlsbad, CA, USA); biotinylated anti-CD59 antibody and FITC-conjugated CD59 were from EXBIO Praha (Vestec, Czech Republic) or BD Biosciences (San Jose, CA, USA); FITC-conjugated CD71 antibody from BD Biosciences.

In all experiments, streptavidin-/antibody-conjugated QD particles were spin at 5,000×g for 5 min and then supernatants were filtered through an 80–100 nm filter at 12,000×g for 5 min prior to use.

### Confocal Microscopy

For single color imaging, Jurkat cells were harvested and pre-fixed by FA of various concentration (0%–10%) in PBS at various temperatures (4°C or 37°C) for 30 min, followed by 3× washes with 5% FBS in PBS. Then fixed cells were stained by biotinylated CTB or biotinylated antibody (anti-CD59 or anti-CD71) at the same temperature as fixation for 20 min. After 3× washes with 5% FBS in PBS, streptavidin-conjugated QD655 dyes were incubated with the cells at corresponding temperature for 20 min, followed by 3× washes with 5% FBS in PBS. Finally, the samples were post-fixed by 2% FA at corresponding temperatures for 30 min and then subject to confocal microscopy on a Carl Zeiss LSM510 Meta5 laser scanning confocal microscope equipped with a 100×1.30 oil-immersion objective. A 405 nm diode laser, LP650 filter, and PMT equipped with the instrument were applied for excitation, filtering and detection, respectively.

Of note, in order to make the confocal data (for statistical use) of different experiments or various groups in a same independent experiment comparable, the confocal settings were always kept identical by reusing previous settings (laser power: 20.8%; pinhole Φ = 1.00 airy units; detector gain = 940; frame size: 1024; line step: 1; scan speed: 7; data depth: 8 bit; mode: line; method: mean; scan number: 8: Zoom: ∼7 for single-cell images).

For two-color confocal imaging in colocalization or crosslinking experiments, similar processes were performed, except that all steps were done at 4°C and only 2% FA was used for cell fixation, and that biotinylated CTB in CTB-crosslinked group, biotinylated CD59 in CD59-crosslinke group, and PHA in PHA-stimulated group were incubated with Jurkat cells for 30 min in CO_2_ incubator prior to fixiaton and QD staining.

### Flow Cytometry

Cell processing was similar to that in confocal microscopic experiments. The difference was that only FITC-conjugated reagents (CTB, anti-CD59, and anti-CD71) were utilized to stain pre-fixed cells. Single-color flow cytometry was conducted on CyAn ADP high-performance research flow cytometer (Dako North America, Carpinteria, CA).

### NSOM Imaging

The information on the NSOM instrument and imaging has been described previously[14], [15]. As we always did, each cell sample was divided into two fractions after pre-fixation, immunostaining, and post-fixation, one of which was used for confocal microscopic observation for confirming a good staining status, and the other one of which was for NSOM imaging. For NSOM imaging, the fixed and stained cells were washed twice by double distilled water. A drop of the cell suspension was deposited onto the 0.1% poly-L-lysine (Sigma-Aldrich, St Louis, MO)-coated fresh coverslips. After air drying for 1 hour, the cells were subjected to Aurora 3 NSOM (Veeco, Santa Barbara, CA). In the study, continuous wave semiconductor laser (Coherent, Cube, 404 nm), 50 nm-aperture NSOM probes, 650/40 nm bandpass filter for fluorescent QD655 and 605/40 nm bandpass filter for fluorescent QD605, and avalanche photon detector (SPCM-AQR-14; PerkinElmer, Vaudreuil, QC) were utilized.

### Data processing and statistics

The data processing has been described previously[15]. Statistic analyses were performed using Student *t* test. It was regarded as significant difference when P<0.05.

## Supporting Information

Figure S1 Confocal microscopy visualized the polish of lipid rafts or the damage of cells due to the cholesterol depletion by M βCD in cell membranes. Jurkat cells were treated without or with 10 or 25 mM M βCD at 37°C for 30 min prior to cell fixation and surface staining of GM1. Upon 10 mM M βCD treatment that was widely used for cholesterol-depletion experiments, a few cells were dead and degraded; the plasma membranes of some cells were partially damaged and the membrane boundaries became indistinct; on the cells with entire plasma membrane, most confocal-resolved microdomains dramatically disassemblied or even disappeared. Upon 25 mM M βCD treatment, the damage became worst: there were full of cell debris in the cell solution, and the dyes entered into the cells that already have no entire plasma membranes.
(1.24 MB TIF)

Figure S2 Two-color confocal microscopy visualizes the colocalization of GM1 and CD59 microdomains. The colocalization (merged color: pink) of GM1 microdomains (pseudocolor: blue; streptavidin-conjugated QD605) with CD59 microdomains (red; goat anti-mouse IgG-conjugated QD655) not CD71 microdomains (red in the first panel: goat anti-mouse IgG-conjugated QD655; green in the 2nd and 4th panels: FITC) was observed on Jurkat T cells treated without or with GM1-crosslinking or CD59-crosslinking prior to cell fixation. Upon PHA stimulation for 30 min prior to fixation, both CD59 and CD71 microdomains colocalized with GM1 domains. All cells were fixed by 2% formaldehyde at 4°C prior to staining in no-treatment group or after crosslinking/stimulating in GM1-linked, CD59-linked, and PHA-stimulated groups, and a second-round fixation was performed after staining.
(2.43 MB TIF)
