# Supplementary figures and images for: Cold Induces Micro- and Nano-Scale Reorganization of Lipid Raft Markers at Mounds of T-Cell Membrane Fluctuations
Source: PLoS One. 2009 Apr 30;4(4):e5386. doi: 10.1371/journal.pone.0005386 (PMC2671402; doi:10.1371/journal.pone.0005386)

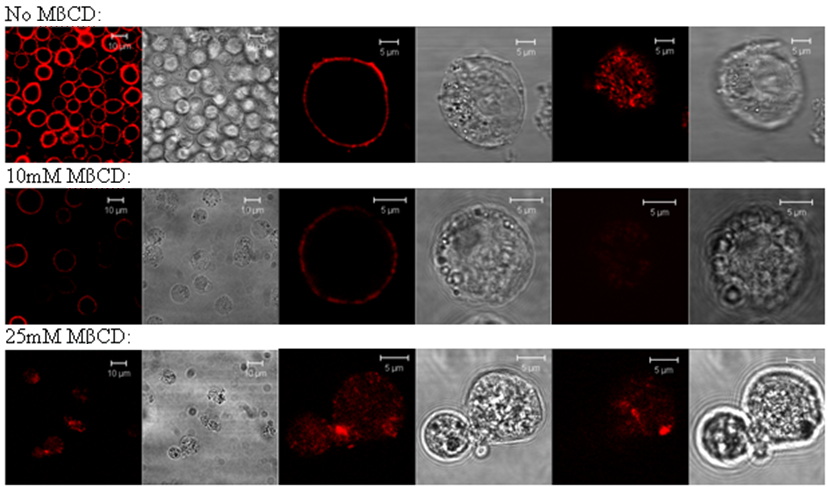

Supplement: Figure S1 — Confocal microscopy visualized the polish of lipid rafts or the damage of cells due to the cholesterol depletion by M βCD in cell membranes. Jurkat cells were treated without or with 10 or 25 mM M βCD at 37°C for 30 min prior to cell fixation and surface staining of GM1. Upon 10 mM M βCD treatment that was widely used for cholesterol-depletion experiments, a few cells were dead and degraded; the plasma membranes of some cells were partially damaged and the membrane boundaries became indistinct; on the cells with entire plasma membrane, most confocal-resolved microdomains dramatically disassemblied or even disappeared. Upon 25 mM M βCD treatment, the damage became worst: there were full of cell debris in the cell solution, and the dyes entered into the cells that already have no entire plasma membranes. (1.24 MB TIF) [file pone.0005386.s001.tif]

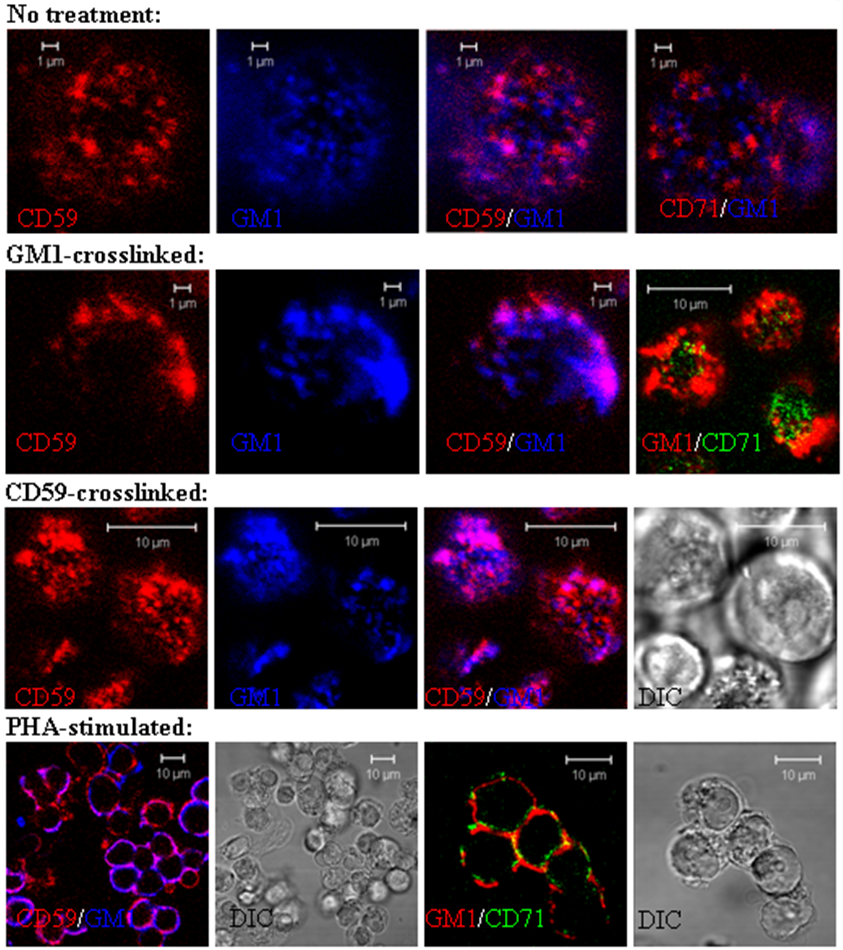

Supplement: Figure S2 — Two-color confocal microscopy visualizes the colocalization of GM1 and CD59 microdomains. The colocalization (merged color: pink) of GM1 microdomains (pseudocolor: blue; streptavidin-conjugated QD605) with CD59 microdomains (red; goat anti-mouse IgG-conjugated QD655) not CD71 microdomains (red in the first panel: goat anti-mouse IgG-conjugated QD655; green in the 2nd and 4th panels: FITC) was observed on Jurkat T cells treated without or with GM1-crosslinking or CD59-crosslinking prior to cell fixation. Upon PHA stimulation for 30 min prior to fixation, both CD59 and CD71 microdomains colocalized with GM1 domains. All cells were fixed by 2% formaldehyde at 4°C prior to staining in no-treatment group or after crosslinking/stimulating in GM1-linked, CD59-linked, and PHA-stimulated groups, and a second-round fixation was performed after staining. (2.43 MB TIF) [file pone.0005386.s002.tif]
